# Effects of Moxibustion Stimulation on the Intensity of Infrared Radiation of Tianshu (ST25) Acupoints in Rats with Ulcerative Colitis

**DOI:** 10.1155/2012/704584

**Published:** 2012-11-20

**Authors:** Xiaomei Wang, Shuang Zhou, Wei Yao, Hua Wan, Huangan Wu, Luyi Wu, Huirong Liu, Xuegui Hua, Peifeng Shi

**Affiliations:** ^1^Key Laboratory of Acupuncture-Moxibustion and Immunological Effects, Shanghai University of Traditional Chinese Medicine, Shanghai 200030, China; ^2^Department of Traditional Chinese Medicine, The Second Military Medical University, Shanghai 200433, China; ^3^Department of Mechanics and Engineering Science, Fudan University, Shanghai 200433, China; ^4^Division of Surgery of Chinese Medicine, Shuguang Hospital, Shanghai University of Traditional Chinese Medicine, Shanghai 201203, China

## Abstract

ST25 is a key acupoint used in the treatment of ulcerative colitis by moxibustion stimulation, but the biophysical mechanism underlying its effects is still unknown. The aim of the present study was to explore the biophysical properties of ST25 acupoint stimulated by moxibustion in a rat model of ulcerative colitis. The infrared radiation intensity of fourteen wavelengths of ST25 showed significant differences between the normal and model control groups. The intensity of infrared radiation of forty wavelengths showed significant differences compared with the corresponding control points in normal rats. The intensity of infrared radiation of twenty-eight wavelengths showed significant differences compared with the corresponding control points in model rats. The intensity of infrared radiation of nine wavelengths in the herb-partition moxibustion group, eighteen wavelengths in the ginger-partition moxibustion group, seventeen wavelengths in the garlic-partition moxibustion group, and fourteen wavelengths in the warming moxibustion group of the left ST25 showed significant differences compared with that of the model control group. For the right-hand-side ST25, these values were 33, 33, 2, and 8 wavelengths, respectively. This indicated that one possible biophysical mechanism of moxibustion on ST25 in ulcerative colitis model rats might involve changes in the intensity of infrared radiation of ST25 at different wavelengths.

## 1. Introduction

The human body is a biological heater that emits infrared rays. Infrared rays can be easily absorbed by objects and penetrate deep into the tissues, where they are transformed into internal energy. The human body is both a source and absorber of infrared radiation [1, 2]. The reported research indicates a significant difference between the infrared radiation patterns of healthy persons and ill ones [3–5]. Infrared radiation can be absorbed if acupuncture points are stimulated by moxibustion [6, 7]. 

Moxibustion is a reputable alternative and complementary therapy with a history of use in eastern countries spanning many thousands of years. Its therapeutic effects depend on meridians and acupoints of the human body [8–11]. Over the past few years, research teams have used thermal infrared imaging to study the patterns of infrared radiation on the surface of the human body along the meridian channel [12, 13]. Studies have found that the infrared radiation spectra of the acupuncture points specifically related to disease changes under disease conditions. In certain morbid conditions of the human body, the infrared radiation of acupoints can differ significantly on the heart meridian and pericardium meridian in patients with coronary artery diseases [14–16]. Patients with chronic stomachaches are distinguished by the patterns of infrared radiation at the stomach Shu (BL21) and Zusanli (ST36) acupoints [17, 18]. Studies have shown a correlation between the meridians and viscera, and the meridian function is closely related to infrared transmission inside and outside of human body [19, 20]. Research has shown that the radiation spectra of moxibustion with ginger, garlic, and monkshood cake were identical to those of healthy human bodies but different from those of bodies subjected to moxa-moxibustion [21]. The acupoints and their infrared resonance radiation patterns have been shown to play an important role in indirect moxibustion stimulation [22, 23]. This indicates that there may be an internal link between moxibustion, acupoints, and meridians that involves infrared transmission.

Tianshu (ST25) points are key acupoints in stomach channel of foot yangming, and moxibustion is an intrinsic part of traditional Chinese medicine (TCM). In clinical practice, acupuncturists apply moxibustion at the specific acupuncture points to treat the corresponding diseases of the viscera; this treatment has been shown to be effective. Research has indicated that moxibustion can modulate gastrointestinal functions [24]. Acupuncture and moxibustion at ST25 can alleviate symptoms and improve quality of life in patients with ulcerative colitis (UC) [25–27]. They have also been shown to improve immune function in UC model rats [28–30]. Our previous study indicated that both Hegu (LI4) and Shangjuxu (ST37) involve changes in infrared radiation spectra in UC patients with intestinal lesions [31]. However, the biophysical mechanism of ST25 stimulated by moxibustion in UC remains unknown.

The present study was performed to establish an UC model in rats, to investigate the intensity of infrared radiation of ST25 stimulated by moxibustion, and to explore the biophysical properties of ST25 acupoint in UC model rats.

## 2. Materials and Methods

### 2.1. Experimental Animals and Model Establishment

Sixty male Sprague-Dawley (SD) rats (weighing 140 ± 20 g) were obtained from the Experimental Animal Center of Shanghai University of Traditional Chinese Medicine. Five male rats were housed in a cage at room temperature (22 ± 2°C) and 60 ± 5% humidity. All rats were performed in strict accordance with the National Institutions of Health Guide for the Care and Use of Laboratory Animals. This study was approved by the Ethics Committee of Yueyang Hospital, which is affiliated with Shanghai University of Traditional Chinese Medicine, China. After three days, the 60 rats were randomly divided into the following experimental groups: normal (*n* = 10), model (*n* = 10), herb-partition moxibustion (HPM) (*n* = 10), ginger-partition moxibustion (GPM) (*n* = 10), garlic-partition moxibustion (GLM) (*n* = 10), and warming moxibustion (WM) (*n* = 10).

The UC rat model was established using an immunological method and local stimulation [32]. In brief, fresh human colonic mucosa was obtained from surgical colonic specimens, homogenized in normal saline, and centrifuged for 30 min at 3000 rpm. The supernatant, containing antigens released from colon of UC patients, was diluted to an appropriate protein concentration and mixed with Freund's adjuvant (Shanghai Chemical Reagent Company, Shanghai, China). One milliliter of the antigen-adjuvant mixture containing a total of 6 mg protein was injected into the front footpad of each rat on day 0. After this initial dose, the same mixture was then injected into the rear footpad, dorsa, inguina, and abdominal cavities on days 10, 17, 24, and 31, respectively. On day 38, in order to stimulate the colonic immune response, rats were anesthetized intraperitoneally with 2% pentobarbital sodium (30 mg/kg) and a 3 mL enema of 3% formalin was administered for 1 hour, then washed with saline, and a 2 mL enema of antigen fluid (without Freund's adjuvant) lasting 2 hours was performed. One rat in model group died of intestinal perforation.

The establishment of the UC rat model was confirmed using hematoxylin-eosin(HE) for pathological observation.

### 2.2. Moxibustion Stimulation

After the UC model was established in the rats, four moxibustion stimulations were performed during days 39–46 in the moxibustion groups, and anesthesia was not applied. Rat holders (Beijing Jinuotai Technology Development Co., Ltd. China) were used to hold the animals in position. 

**Figure 1:**
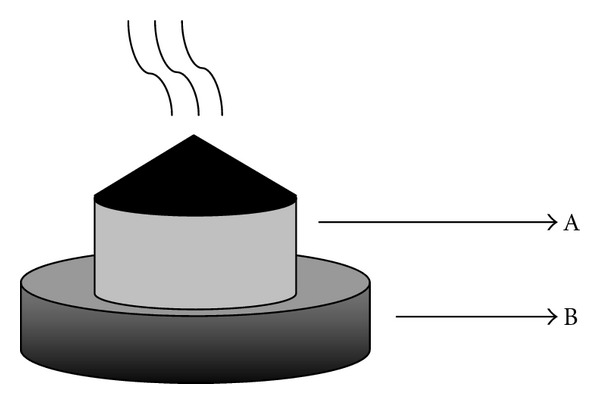
Sketch of herb-partition moxibustion. A: Moxa segment. B: Herb-tablet.

HPM (*n* = 10): as indicated in Figure 1, moxa cones (Figure 1A) (0.5 cm in diameter and 0.6 cm high) (Nanyang Hanyi Moxa Co., Ltd. China) were placed vertically on a medicinal formula (diameter 0.5 cm, height 0.3 cm) composed of radix aconite, cortex, radix, carthami, and salviae miltiorrhizae (Figure 1B). The medicinal formula was then placed on ST25 acupoints [33, 34]. The moxa cones were then ignited, and each acupoint was treated twice lasting 15 min.

GPM (*n* = 10): as indicated in Figure 1, moxa cones were placed vertically on a fresh ginger (diameter 0.5 cm, high 0.3 cm) instead of medicinal formula (Figure 1B). The fresh ginger was then placed on ST25 acupoints. The moxa cones were then ignited, and each acupoint was treated twice lasting 15 min. 

GLM (*n* = 10): as indicated in Figure 1, moxa cones were placed vertically on a fresh garlic (diameter 0.5cm, height 0.3cm) instead of medicinal formula (Figure 1B). The fresh garlic was then placed on ST25 acupoints. The moxa cones were then ignited, and each acupoint was treated twice lasting 15 min.

**Figure 2:**
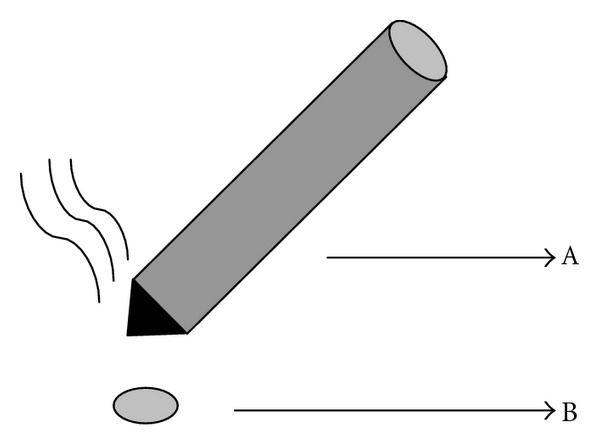
Sketch of warm moxibustion. A: Moxa stick. B: Acupoint.

WM (*n* = 10): as indicated in Figure 2, the moxa stick (Figure 2A) (diameter 0.5 cm, length 20 cm) was ignited and hung 2 cm above the ST25 acupoints (Figure 2B). Each acupoint was treated once for 10 min. 

NC (*n* = 10): no treatment. The same fixation as the moxibustion groups was administered.

MC (*n* = 10): no treatment. The same fixation as the moxibustion groups was administered.

All treatments were repeated once daily for a total of 14 d [35].

### 2.3. Morphological Observation of Colon Samples

After the infrared measurements finished, all rats were killed by cervical dislocation. The samples were collected from the descending colon (5 cm above the anus) and cleaned with normal saline. General morphology was then assessed and scored. The samples were fixed in 10% formalin, dehydrated, embedded in paraffin, and sectioned into 4 *μ*m thick slices. These sections were then stained with hematoxylin-eosin for pathological observation [36].

### 2.4. Detection of the Intensity of Infrared Radiation

A hypersensitivity PHE201 infrared spectrum analyzer was used to detect the intensity of infrared radiation on bilateral ST25 and negative control points (0.5 cm from ST25) of experimental rats. The 59 measurement wavelengths range from 1.5–16 *μ*m [1, 22].

**Figure 3:**
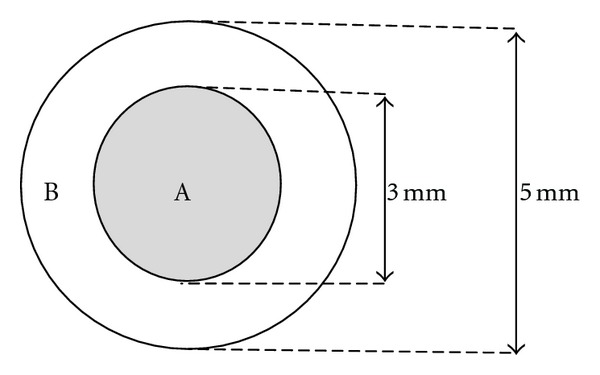
Sketch of points of detection and moxibustion stimulation. A: Point of detection. B: Point of moxibustion stimulation.

In the morning of the next day after moxibustion treatments finished, all rats were brought into a darkroom. None were anesthetized. The room was maintained at 22 ± 3°C room temperature and 55 ± 10% humidity and it had no obvious airflow, strong noises, or electromagnetic fields. Thirty minutes later, the acupoints were defatted using 75% alcohol. An analyzer probe (diameter 3 mm) (Figure 3) was gently placed on the acupoints. The analyzer was initialized to scan the wavelengths from 1.5 to 6 *μ*m. The analyzer automatically recorded the intensity of infrared radiation at a total of 59 detection points every 0.25 *μ*m.

### 2.5. Statistical Analysis

All data were analyzed using SPSS 11.0 statistical software (SPSS Inc., USA). All data are expressed as mean ± SD for normally distributed continuous variables. Rank-sum testing was used to compare the four moxibustion groups with normal and model control groups. The pairing rank-sum test was used to compare the right- and left-hand-side acupoints. *P* < 0.05 was considered statistically significant.

## 3. Results

### 3.1. Morphological Observation of UC Rat Model [37]

**Figure 4:**
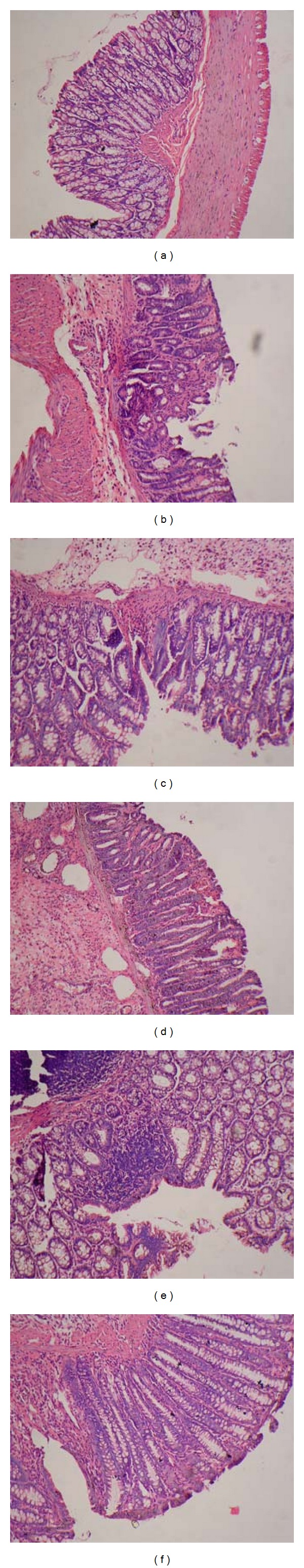
Morphological observation of UC rat model. (a) Normal group. (b) Model group. (c) Herb-partition moxibustion group. (d) Ginger-partition moxibustion group. (e) Garlic-partition moxibustion group. (f) Warming moxibustion group.

As shown in Figure 4, the NC group showed complete colonic mucosa epithelium and regular colonic gland with inconspicuous inflammatory cell infiltration. The colonic mucosa and mucosa villi were damaged or missing in the UC model group, and large numbers of mononuclear cells and macrophages appeared in the mucosa or submucosa. There was also more congestion, edema, and ulceration than in the normal group. However, moxibustion stimulation with herbs, ginger, garlic, and moxa sticks decreased the inflammatory cell infiltration and improved the condition of the colonic mucosa and mucosa villi. Only slight submucosal edema and inflammatory cell infiltration were observed in the treated groups, and the colonic mucosa epithelium and the colonic gland were more regularly arranged in all treated groups except the mild moxibustion group than in the model group. New epithelial cells were observed, covering the ulcers. This indicates that moxibustion treatment can inhibit inflammatory cell infiltration under UC conditions and induce recovery of these ulcers in the colon tissue.

### 3.2. Infrared Spectrum Characteristic of ST25 and Control Points

Each curve showed the average infrared radiation of ST25 in rats from each group. The intensity of infrared radiation of ST25 showed significant individual and intergroup differences, but each group showed the same spectral peak. The first peak value appeared at about 10 *μ*m, and the second, which was weaker than the first, appeared at about 13.75 *μ*m. 

### 3.3. Intensity of Infrared Radiation at ST25 and Control Points in Normal Rats (Table 1)

NC group rats showed significant differences in the intensity of infrared radiation of the left ST25 and control point at 4.00, 4.75, 5.00, 5.50, 6.25, 6.50, 6.75, 15.50, and 16.00 *μ*m (*P* < 0.05) and extremely significant differences at 5.25 *μ*m (*P* < 0.01). On the right side, the intensity of infrared radiation of ST25 differed significantly from control point at 8.00, 8.25, 8.50, 9.00, 9.25, 9.5, 9.75, 10.00, 10.25, 10.50, 11.00, 12.00, 12.75, 13.00, 13.50, 13.75, 14.00, 14.25, 14.5, 14.75, 15.00, 15.25, and 15.5 *μ*m (*P* < 0.05) and differed extremely significantly at 10.75, 11.25, 11.50, 11.75, 12.25, 12.50, and 15.75 *μ*m (*P* < 0.01). The intensities of infrared radiation of the left-hand-side ST25 differed significantly from the right-hand-side ST25 at 5.25, 5.50, 6.25, and 16.00 *μ*m (*P* < 0.05).

### 3.4. Intensity of Infrared Radiation at ST25 and Control Points in UC Model Rats (Table 2)

In MC group rats, the intensity of infrared radiation showed significant differences across the left ST25 and control point at 1.50, 4.75, and 5.00 *μ*m (*P* < 0.05). On the right-hand side, the intensities of infrared radiation of ST25 differed from control point at 5.00, 7.00, 8.00, 8.75, 9.75, 10.25, 10.50, 10.75, 11.75, 12.00, 12.50, 12.75, 13.00, 13.25, 13.50, 13.75, and 14.00 *μ*m (*P* < 0.05) and differed very significantly at 7.25, 7.75, 8.25, 8.50, 9.00, 9.25, 9.50, and 10.00 *μ*m (*P* < 0.01). However, there was no difference between the intensity of infrared radiation at the left and right ST25.

### 3.5. Intensities of Infrared Radiation of the Left ST25 after Different Moxibustion Stimulations (Table 3)

There were significant differences in the intensity of infrared radiation of the left ST25 between the NC and the MC groups at 3.00, 3.75, 4.75, 5.75, 6.00 *μ*m (*P* < 0.05) and at 2.50, 2.75, 5.25 *μ*m (*P* < 0.01).

After different types of moxibustion stimulation, the intensity of infrared radiation of the left ST25 of the HPM group was significantly different from that of the MC group at 2.50, 3.00, 10.25, 10.50, 11.00, 11.25, and 11.50 *μ*m (*P* < 0.05) and very significantly different at 2.75 and 10.75 *μ*m (*P* < 0.01). The differences between GPM group and MC group were significant at 2.50, 7.50, 8.00, 8.25, 8.50, 9.00, 9.25, 9.75, 10.00, 10.25, 10.75, 11.00, 11.25, 11.50, 11.75, and 14.00 *μ*m (*P* < 0.05) and very significant at 2.75, 9.25, and 10.50 *μ*m (*P* < 0.01). The differences between GLM group and MC group were significant at 2.75, 3.00, 7.50, 8.00, 8.50, 8.75, 9.00, 9.25, 9.50, 9.75, 10.00, 10.25, 10.50, 10.75, 11.50, and 12.00 *μ*m (*P* < 0.05) and very significant at 8.25 *μ*m (*P* < 0.01). The intensity of infrared radiation of the left ST25 in the WM group was significantly different from those of the MC group at 2.50, 10.00, 10.25, 11.00, 11.25, 11.75, 12.00, 13.75, and 14.25 *μ*m (*P* < 0.05) and very significantly different at 2.75, 10.50, 10.75, 11.50, and 14.00 *μ*m (*P* < 0.01). 

For the intensity of infrared radiation of left ST25, eight wavelengths showed a statistically significant difference between the NC and MC groups. After moxibustion stimulation, there were 9 wavelengths in the HPM group, 18 wavelengths in the GPM group, 17 wavelengths in the GLM group, and 14 wavelengths in the WM group that showed statistically significant differences from the MC group.

### 3.6. Intensity of Infrared Radiation of the Right ST25 after Different Moxibustion Stimulations (Table 4)

The intensity of infrared radiation of the right ST25 showed significant differences between the NC and MC groups at 1.75, 4.00, 4.25, and 4.75 *μ*m (*P* < 0.05) and at 2.75 and 3.00 *μ*m (*P* < 0.01). 

After different types of moxibustion stimulation, the intensity of infrared radiation of the right ST25 of the HPM group was significantly different from that of the MC group at 2.25, 7.25, 7.50, 7.75, 8.00, 8.50, 8.75, 9.50, 12.50, 13.25, and 14.25 *μ*m (*P* < 0.05) and very significantly different at 1.75, 2.75, 3.00, 8.25, 9.00, 9.25, 9.75, 10.00, 10.25, 10.50, 10.75, 11.00, 11.25, 11.50, 11.75, 12.00, 12.25, 12.75, 13.00, 13.50, 13.75, and 14.00 *μ*m (*P* < 0.01). The differences between GPM group and MC group were significant at 13.50, 14.75, and 15.00 *μ*m (*P* < 0.05) and very significant at 3.00, 7.00, 7.25, 7.50, 7.75, 8.00, 8.25, 8.50, 8.75, 9.00, 9.25, 9.50, 9.75, 10.00, 10.25, 10.50, 10.75, 11.00, 11.25, 11.50, 11.75, 12.00, 12.25, 12.50, 12.75, 13.00, 13.75, 14.00, 14.25, and 14.50 *μ*m (*P* < 0.01). The differences between the GLM group and MC group were significant at 3.00 and 8.25 *μ*m (*P* < 0.05). The intensity of infrared radiation of the right ST25 in the WM group and MC group was significantly different at 1.75, 3.00, 10.25, 10.50, 10.75, 11.00, and 14.00 *μ*m (*P* < 0.05) and very significantly different at 2.75 *μ*m (*P* < 0.01). 

For the intensity of infrared radiation of the right ST25, 6 wavelengths showed a statistically significant difference between the NC and MC groups. After moxibustion stimulation, 33 wavelengths in the HPM group, 33 wavelengths in the GPM group, 2 wavelengths in the GLM group, and 8 wavelengths in the WM group showed statistically significant differences from the MC group.

## 4. Discussion

In traditional Chinese medicine, acupoints are the points of infusion and transmission of qi and blood of viscus and meridians onto the surface of the human body and the windows of connection between the human body and its surroundings. In this way, infrared radiation and the transmission characteristics of acupoints can indicate the state of infusion of qi and blood and of the body's physiological response to pathological changes in the visceral organs.

Traditional Chinese medicine, which includes moxibustion, has been developing for more than 2500 years, and it has a long history of being used to prevent and treat diseases in Eastern cultures. Moxibustion therapy is a stimulation of acupoints by the burning of moxa. Moxibustion therapy includes both direct moxibustion and indirect moxibustion. Indirect moxibustion is a relatively common clinical therapy, and it includes herb-partition moxibustion, ginger-partition moxibustion, garlic-partition moxibustion, and other types of moxibustion. Moxibustion, through its warming effect and medicinal properties, improves the flow of both qi and blood through the acupoints and meridians, regulating the functions of viscus and organs to prevent and treat illnesses. Studies have indicated that moxibustion therapy can improve human immunity and regulate digestive function [24, 26, 38]. However, the biophysical mechanism underlying moxibustion and the role of acupoints in this process are unknown.

Several studies have indicated that infrared radiation is natural on the surface of the human body [39–41]. Ever since French scientist J. Borsarello photographed the infrared thermogram to show meridians and acupoints in human body in 1970, researchers at home and abroad have carried out many studies regarding the infrared thermography characteristics of the human body. Progress has been made on the meridian phenomenon, the characteristics of acupoints, acupuncture technique, and the therapeutic effects of acupuncture. Research has shown that the body emits infrared radiation along specific tracks and that acupuncture may cause changes in skin temperature [42–48]. Reinforcing acupuncture techniques can increase skin temperature and reducing methods can decrease skin temperature [49–55]. The duration of retaining needle and the warming effects of acupuncture has certain relation [56]. The body may experience acupoint temperature imbalances during pathologic states [57]. However, the human body shows individual differences with respect to infrared radiation, and the infrared radiation is readily influenced by physiological factors such as sweating and nervousness and by environment factors such as atmospheric convection. In this way, data concerning skin temperature and changes in it are questionable and cannot alone be used to determine the mechanism underlying acupoint infrared radiation. 

In the theory of traditional Chinese medicine, the Tianshu (ST25) acupoint is located at the stomach channel of foot yangming. It is the frontmost Mu point of the large intestine channel of hand yangming and the hub of ascending lucidity and descending turbidity in the human body. It can recruit qi and blood along the stomach channel and transmit both to the large intestinal channel. Therefore, ST25 is closely related to the gastrointestinal tract for the regulation of gastrointestinal function [33, 58]. Because it has antidiarrheal and cathartic effects, it is used to treat gastrointestinal diseases in clinic practice. Previous studies have indicated that ST25 plays an important role in the treatment of gastrointestinal diseases [59–64]. Acupuncture and moxibustion stimulation at ST25 can activate mast cell degranulation and downregulate colonic epithelial cell apoptosis in colitis model rats [59, 60]. They have also been shown to have analgesic effects in rats subjected to chronic visceral hypersensitivity [61, 62]. They regulate gastrointestinal function in human patients [63, 64]. In the present study, the intensity of infrared radiation of bilateral ST25 and the control points in UC rats were measured using a hypersensitivity PHE201 infrared spectrum analyzer [1, 22]. Results indicated that the intensities of infrared radiation of ST25 in the NC and MC groups differed from control points and that the difference on the right side was more marked than on the left side. However, there was little difference between the right and left ST25. This indicated the specificity of ST25 acupoint relative to control points. The intensities of infrared radiation of ST25 showed differences between the NC and MC groups and between the HPM and MC groups. However, further research must be performed using larger samples. Some studies have indicated that the near-infrared radiation from the moxa moxibustion burning process can energize hydrogen bonds inside the acupoints and allow them to absorb the stimulated resonance. They would then transmit energy to the cells through the nerve-fluid system [38]. This also showed that moxibustion stimulation could improve the condition of colon tissue in UC model rats, indicating that the infrared radiation of acupoints and moxibustion treatment has a biophysical foundation. Other studies have reported that the infrared radiation spectra of radix aconite-partition moxibustion and acupoints are amazingly consistent. The therapeutic effects of indirect moxibustion include the physical effects of moxibustion thermal radiation, the therapeutic effect of moxa and partition, and the resonance of the infrared radiation of indirect moxibustion and acupoints [65, 66].

## 5. Conclusion

Acupoints are specific, and moxibustion, especially thing-partition moxibustion, can regulate the running state of both qi and blood in acupoints. In this way, they can be used to treat illness by influencing the infrared physical effect of the corresponding acupoints. An in-depth study of these effects would reveal the biophysical properties of these acupoints, the pathologic characteristics of UC, and the biophysical mechanisms underlying the therapeutic effects of moxibustion. It may also provide new research methods that can be used to diagnose illness and explore the effects of acupuncture and moxibustion stimulation.

## Figures and Tables

**Table 1:** Intensity of infrared radiation of ST25 and the control points in normal rats (mean ± SD).

WL (*μ*m)	*N*	L-ST25	L-ST25-N	R-ST25	R-ST25-N
1.50	10	0.032 ± 0.027	0.041 ± 0.018	0.035 ± 0.024	0.031 ± 0.013
1.75	10	−0.002 ± 0.022	0.004 ± 0.027	0.001 ± 0.022	−0.005 ± 0.030
2.00	10	0.002 ± 0.025	−0.001 ± 0.023	−0.002 ± 0.024	−0.004 ± 0.027
2.25	10	−0.003 ± 0.021	0.008 ± 0.019	0.005 ± 0.020	0.007 ± 0.023
2.50	10	−0.003 ± 0.020	0.005 ± 0.025	0.005 ± 0.029	−0.003 ± 0.024
2.75	10	−0.004 ± 0.022	0.005 ± 0.023	0.001 ± 0.022	−0.002 ± 0.031
3.00	10	−0.006 ± 0.019	0.007 ± 0.034	0.002 ± 0.025	−0.002 ± 0.030
3.25	10	0.001 ± 0.019	0.007 ± 0.019	0.005 ± 0.023	0.004 ± 0.026
3.50	10	0.023 ± 0.020	0.035 ± 0.031	0.021 ± 0.028	0.028 ± 0.030
3.75	10	0.019 ± 0.029	0.025 ± 0.020	0.032 ± 0.031	0.036 ± 0.028
4.00	10	0.006 ± 0.023	0.031 ± 0.023^Δ^	0.016 ± 0.015	0.024 ± 0.028
4.25	10	0.037 ± 0.023	0.058 ± 0.020	0.048 ± 0.021	0.060 ± 0.033
4.50	10	0.067 ± 0.018	0.075 ± 0.028	0.068 ± 0.021	0.082 ± 0.032
4.75	10	0.063 ± 0.030	0.094 ± 0.024^Δ^	0.076 ± 0.029	0.092 ± 0.037
5.00	10	0.074 ± 0.020	0.099 ± 0.030^Δ^	0.094 ± 0.025	0.100 ± 0.031
5.25	10	0.079 ± 0.024	0.118 ± 0.026^ΔΔ^	0.106 ± 0.024*	0.123 ± 0.025
5.50	10	0.059 ± 0.023	0.086 ± 0.026^Δ^	0.082 ± 0.022*	0.095 ± 0.026
5.75	10	0.048 ± 0.020	0.064 ± 0.026	0.057 ± 0.017	0.070 ± 0.040
6.00	10	0.066 ± 0.024	0.086 ± 0.028	0.082 ± 0.017	0.103 ± 0.031
6.25	10	0.044 ± 0.023	0.070 ± 0.027^Δ^	0.068 ± 0.026*	0.075 ± 0.026
6.50	10	0.046 ± 0.022	0.078 ± 0.026^Δ^	0.059 ± 0.028	0.077 ± 0.030
6.75	10	0.143 ± 0.026	0.182 ± 0.032^Δ^	0.147 ± 0.038	0.174 ± 0.050
7.00	10	0.224 ± 0.040	0.275 ± 0.032	0.230 ± 0.065	0.274 ± 0.078
7.25	10	0.322 ± 0.055	0.365 ± 0.066	0.341 ± 0.076	0.395 ± 0.087
7.50	10	0.374 ± 0.058	0.422 ± 0.070	0.393 ± 0.064	0.455 ± 0.081
7.75	10	0.404 ± 0.066	0.458 ± 0.073	0.428 ± 0.077	0.493 ± 0.090
8.00	10	0.473 ± 0.075	0.522 ± 0.094	0.477 ± 0.087	0.563 ± 0.104^Δ^
8.25	10	0.480 ± 0.080	0.541 ± 0.079	0.509 ± 0.086	0.606 ± 0.118^Δ^
8.50	10	0.494 ± 0.077	0.555 ± 0.094	0.519 ± 0.095	0.609 ± 0.103^Δ^
8.75	10	0.571 ± 0.078	0.642 ± 0.114	0.590 ± 0.105	0.684 ± 0.130
9.00	10	0.660 ± 0.100	0.744 ± 0.122	0.686 ± 0.122	0.808 ± 0.152^Δ^
9.25	10	0.690 ± 0.099	0.794 ± 0.140	0.721 ± 0.134	0.865 ± 0.157^Δ^
9.50	10	0.750 ± 0.096	0.856 ± 0.149	0.791 ± 0.133	0.923 ± 0.161^Δ^
9.75	10	0.809 ± 0.118	0.911 ± 0.161	0.850 ± 0.138	0.997 ± 0.163^Δ^
10.00	10	0.846 ± 0.123	0.953 ± 0.162	0.892 ± 0.150	1.043 ± 0.176^Δ^
10.25	10	0.835 ± 0.135	0.936 ± 0.141	0.874 ± 0.138	1.047 ± 0.173^Δ^
10.50	10	0.739 ± 0.140	0.825 ± 0.138	0.768 ± 0.117	0.933 ± 0.157^Δ^
10.75	10	0.655 ± 0.121	0.732 ± 0.120	0.679 ± 0.118	0.834 ± 0.144^ΔΔ^
11.00	10	0.580 ± 0.115	0.658 ± 0.108	0.620 ± 0.110	0.753 ± 0.123^Δ^
11.25	10	0.548 ± 0.094	0.613 ± 0.100	0.556 ± 0.094	0.683 ± 0.121^ΔΔ^
11.50	10	0.496 ± 0.090	0.549 ± 0.091	0.501 ± 0.093	0.627 ± 0.123^ΔΔ^
11.75	10	0.433 ± 0.086	0.488 ± 0.090	0.448 ± 0.085	0.559 ± 0.098^ΔΔ^
12.00	10	0.407 ± 0.067	0.466 ± 0.073	0.427 ± 0.072	0.510 ± 0.080^Δ^
12.25	10	0.410 ± 0.057	0.466 ± 0.065	0.430 ± 0.061	0.518 ± 0.086^ΔΔ^
12.50	10	0.387 ± 0.064	0.445 ± 0.070	0.420 ± 0.074	0.511 ± 0.088^ΔΔ^
12.75	10	0.359 ± 0.077	0.409 ± 0.082	0.385 ± 0.070	0.460 ± 0.085^Δ^
13.00	10	0.362 ± 0.065	0.415 ± 0.058	0.372 ± 0.067	0.449 ± 0.075^Δ^
13.25	10	0.462 ± 0.054	0.529 ± 0.088	0.465 ± 0.092	0.534 ± 0.109
13.50	10	0.587 ± 0.073	0.661 ± 0.110	0.605 ± 0.101	0.699 ± 0.124^Δ^
13.75	10	0.590 ± 0.092	0.660 ± 0.119	0.616 ± 0.106	0.735 ± 0.133^Δ^
14.00	10	0.509 ± 0.113	0.560 ± 0.107	0.531 ± 0.100	0.651 ± 0.123^Δ^
14.25	10	0.426 ± 0.095	0.485 ± 0.085	0.467 ± 0.098	0.561 ± 0.108^Δ^
14.50	10	0.363 ± 0.076	0.416 ± 0.072	0.399 ± 0.083	0.487 ± 0.092^Δ^
14.75	10	0.258 ± 0.072	0.299 ± 0.058	0.286 ± 0.066	0.356 ± 0.060^Δ^
15.00	10	0.205 ± 0.058	0.240 ± 0.040	0.227 ± 0.059	0.278 ± 0.054^Δ^
15.25	10	0.177 ± 0.050	0.200 ± 0.031	0.190 ± 0.043	0.226 ± 0.038^Δ^
15.50	10	0.178 ± 0.033	0.212 ± 0.026^Δ^	0.201 ± 0.041	0.241 ± 0.033^Δ^
15.75	10	0.172 ± 0.037	0.198 ± 0.026	0.192 ± 0.029	0.234 ± 0.042^ΔΔ^
16.00	10	0.137 ± 0.038	0.168 ± 0.034^Δ^	0.170 ± 0.026*	0.195 ± 0.036

WL: wavelength, L-ST25: left ST25, L-ST25-N: left ST25 control point, R-ST25: right ST25, R-ST25-N: right control point. The intensity of the left-hand-side infrared radiation of ST25 in normal rats is significantly different from that of right-hand-side ST25 (*P < 0.05, **P < 0.01). The intensity of infrared radiation of ST25 in normal rats is significantly different from that of the ipsilateral control point (^Δ^ *P* < 0.05, ^ΔΔ^ *P* < 0.01).

**Table 2:** Intensity of infrared radiation of ST25 and the control points in UC model rats (mean ± SD).

WL (*μ*m)	N	L-ST25	L-ST25-N	R-ST25	R-ST25-N
1.50	9	−0.003 ± 0.035	0.023 ± 0.025^Δ^	0.009 ± 0.014	0.010 ± 0.022
1.75	9	0.005 ± 0.025	0.013 ± 0.024	0.021 ± 0.028	0.016 ± 0.021
2.00	9	0.017 ± 0.030	0.026 ± 0.021	0.007 ± 0.025	0.020 ± 0.030
2.25	9	0.008 ± 0.033	0.016 ± 0.024	0.018 ± 0.026	0.025 ± 0.023
2.50	9	0.022 ± 0.018	0.018 ± 0.023	0.013 ± 0.027	0.022 ± 0.025
2.75	9	0.024 ± 0.019	0.025 ± 0.020	0.022 ± 0.017	0.028 ± 0.019
3.00	9	0.021 ± 0.023	0.023 ± 0.022	0.029 ± 0.021	0.025 ± 0.020
3.25	9	0.015 ± 0.030	0.027 ± 0.023	0.016 ± 0.024	0.028 ± 0.028
3.50	9	0.030 ± 0.021	0.045 ± 0.027	0.036 ± 0.026	0.052 ± 0.029
3.75	9	0.043 ± 0.017	0.053 ± 0.020	0.039 ± 0.022	0.051 ± 0.030
4.00	9	0.031 ± 0.033	0.040 ± 0.033	0.038 ± 0.036	0.034 ± 0.031
4.25	9	0.059 ± 0.035	0.075 ± 0.036	0.077 ± 0.041	0.071 ± 0.042
4.50	9	0.073 ± 0.018	0.091 ± 0.014	0.074 ± 0.025	0.093 ± 0.028
4.75	9	0.084 ± 0.023	0.107 ± 0.021^Δ^	0.096 ± 0.026	0.103 ± 0.020
5.00	9	0.085 ± 0.016	0.105 ± 0.022^Δ^	0.087 ± 0.019	0.112 ± 0.022^Δ^
5.25	9	0.109 ± 0.041	0.124 ± 0.035	0.121 ± 0.039	0.128 ± 0.030
5.50	9	0.079 ± 0.022	0.101 ± 0.028	0.088 ± 0.030	0.111 ± 0.028
5.75	9	0.070 ± 0.019	0.086 ± 0.028	0.075 ± 0.029	0.084 ± 0.025
6.00	9	0.095 ± 0.028	0.111 ± 0.027	0.102 ± 0.024	0.116 ± 0.024
6.25	9	0.065 ± 0.019	0.078 ± 0.023	0.073 ± 0.034	0.085 ± 0.029
6.50	9	0.062 ± 0.033	0.082 ± 0.035	0.067 ± 0.035	0.084 ± 0.032
6.75	9	0.164 ± 0.028	0.170 ± 0.021	0.165 ± 0.039	0.191 ± 0.037
7.00	9	0.232 ± 0.030	0.246 ± 0.032	0.234 ± 0.042	0.282 ± 0.049^Δ^
7.25	9	0.328 ± 0.049	0.348 ± 0.023	0.334 ± 0.053	0.394 ± 0.049^ΔΔ^
7.50	9	0.359 ± 0.054	0.378 ± 0.046	0.378 ± 0.074	0.452 ± 0.067^Δ^
7.75	9	0.408 ± 0.066	0.414 ± 0.041	0.408 ± 0.078	0.492 ± 0.065^ΔΔ^
8.00	9	0.457 ± 0.075	0.482 ± 0.035	0.472 ± 0.078	0.559 ± 0.076^Δ^
8.25	9	0.474 ± 0.075	0.504 ± 0.055	0.484 ± 0.088	0.586 ± 0.087^ΔΔ^
8.50	9	0.482 ± 0.072	0.506 ± 0.060	0.491 ± 0.081	0.590 ± 0.084^ΔΔ^
8.75	9	0.546 ± 0.088	0.575 ± 0.056	0.569 ± 0.088	0.671 ± 0.095^Δ^
9.00	9	0.635 ± 0.095	0.666 ± 0.072	0.657 ± 0.105	0.792 ± 0.117^ΔΔ^
9.25	9	0.671 ± 0.104	0.705 ± 0.070	0.698 ± 0.102	0.835 ± 0.102^ΔΔ^
9.50	9	0.738 ± 0.114	0.788 ± 0.072	0.772 ± 0.115	0.912 ± 0.113^ΔΔ^
9.75	9	0.778 ± 0.117	0.824 ± 0.090	0.805 ± 0.124	0.946 ± 0.126^Δ^
10.00	9	0.791 ± 0.113	0.834 ± 0.095	0.817 ± 0.131	0.971 ± 0.116^ΔΔ^
10.25	9	0.791 ± 0.117	0.827 ± 0.093	0.814 ± 0.134	0.964 ± 0.118^Δ^
10.50	9	0.676 ± 0.118	0.715 ± 0.100	0.711 ± 0.126	0.852 ± 0.119^Δ^
10.75	9	0.600 ± 0.099	0.642 ± 0.086	0.633 ± 0.115	0.742 ± 0.111^Δ^
11.00	9	0.551 ± 0.097	0.588 ± 0.083	0.573 ± 0.111	0.660 ± 0.097
11.25	9	0.505 ± 0.081	0.549 ± 0.090	0.541 ± 0.107	0.616 ± 0.104
11.50	9	0.456 ± 0.091	0.485 ± 0.078	0.488 ± 0.095	0.567 ± 0.085
11.75	9	0.415 ± 0.086	0.447 ± 0.061	0.433 ± 0.092	0.513 ± 0.069^Δ^
12.00	9	0.377 ± 0.067	0.414 ± 0.045	0.407 ± 0.069	0.474 ± 0.062^Δ^
12.25	9	0.383 ± 0.065	0.430 ± 0.061	0.413 ± 0.070	0.473 ± 0.070
12.50	9	0.385 ± 0.057	0.414 ± 0.047	0.402 ± 0.065	0.473 ± 0.070^Δ^
12.75	9	0.377 ± 0.060	0.396 ± 0.050	0.380 ± 0.069	0.453 ± 0.059^Δ^
13.00	9	0.351 ± 0.074	0.385 ± 0.047	0.365 ± 0.070	0.440 ± 0.061^Δ^
13.25	9	0.447 ± 0.072	0.477 ± 0.050	0.465 ± 0.068	0.548 ± 0.077^Δ^
13.50	9	0.554 ± 0.071	0.590 ± 0.046	0.578 ± 0.091	0.674 ± 0.092^Δ^
13.75	9	0.555 ± 0.092	0.597 ± 0.070	0.580 ± 0.091	0.677 ± 0.097^Δ^
14.00	9	0.454 ± 0.091	0.488 ± 0.073	0.473 ± 0.095	0.564 ± 0.089^Δ^
14.25	9	0.396 ± 0.078	0.444 ± 0.066	0.423 ± 0.086	0.500 ± 0.091
14.50	9	0.351 ± 0.076	0.383 ± 0.070	0.375 ± 0.074	0.444 ± 0.073
14.75	9	0.255 ± 0.048	0.288 ± 0.051	0.267 ± 0.064	0.312 ± 0.053
15.00	9	0.217 ± 0.041	0.238 ± 0.043	0.221 ± 0.050	0.254 ± 0.041
15.25	9	0.185 ± 0.032	0.202 ± 0.030	0.196 ± 0.040	0.215 ± 0.049
15.50	9	0.196 ± 0.035	0.205 ± 0.035	0.201 ± 0.044	0.221 ± 0.041
15.75	9	0.193 ± 0.026	0.211 ± 0.035	0.208 ± 0.037	0.219 ± 0.040
16.00	9	0.158 ± 0.030	0.171 ± 0.033	0.164 ± 0.035	0.180 ± 0.040

WL: wavelength, L-ST25: left ST25, L-ST25-N: left ST25 control point, R-ST25: right ST25, R-ST25-N: right control point. The intensity of infrared radiation of ST25 in model rats was found to be significantly different from that of the ipsilateral control point (^Δ^ *P* < 0.05, ^ΔΔ^ *P* < 0.01).

**Table 3:** Intensity of infrared radiation of left-hand-side ST25 after different moxibustion treatments.

WL (*μ*m)	NC	MC	HPM	GPM	GLM	WM
1.50	−0.008 ± 0.030	−0.003 ± 0.035	0.007 ± 0.015	0.008 ± 0.014	0.009 ± 0.013	0.002 ± 0.011
1.75	−0.002 ± 0.022	0.005 ± 0.025	0.003 ± 0.013	0.009 ± 0.015	0.012 ± 0.023	−0.002 ± 0.009
2.00	0.002 ± 0.025	0.017 ± 0.030	0.012 ± 0.011	0.015 ± 0.012	0.003 ± 0.025	0.006 ± 0.012
2.25	−0.003 ± 0.021	0.008 ± 0.033	0.004 ± 0.018	0.020 ± 0.021	0.006 ± 0.020	0.004 ± 0.016
2.50	−0.003 ± 0.020	0.022 ± 0.018**	0.002 ± 0.016^Δ^	0.004 ± 0.015^Δ^	0.006 ± 0.013	0.006 ± 0.015^Δ^
2.75	−0.004 ± 0.022	0.024 ± 0.019**	−0.001 ± 0.016^ΔΔ^	0.010 ± 0.015	0.007 ± 0.011^Δ^	0.004 ± 0.015^ΔΔ^
3.00	−0.006 ± 0.019	0.021 ± 0.023*	0.003 ± 0.020^Δ^	0.005 ± 0.019	0.001 ± 0.017^Δ^	0.009 ± 0.013
3.25	0.001 ± 0.019	0.015 ± 0.030	0.011 ± 0.017	0.012 ± 0.019	0.017 ± 0.012	0.004 ± 0.023
3.50	0.022 ± 0.020	0.030 ± 0.021	0.025 ± 0.035	0.032 ± 0.020	0.029 ± 0.018	0.017 ± 0.015
3.75	0.019 ± 0.029	0.043 ± 0.017*	0.031 ± 0.020	0.040 ± 0.017	0.036 ± 0.024	0.034 ± 0.012
4.00	0.006 ± 0.023	0.031 ± 0.033	0.023 ± 0.030	0.024 ± 0.017	0.035 ± 0.034	0.034 ± 0.029
4.25	0.037 ± 0.023	0.059 ± 0.035	0.056 ± 0.025	0.058 ± 0.021	0.055 ± 0.031	0.059 ± 0.030
4.50	0.067 ± 0.018	0.073 ± 0.018	0.057 ± 0.032	0.078 ± 0.015	0.072 ± 0.027	0.058 ± 0.024
4.75	0.063 ± 0.030	0.084 ± 0.023*	0.071 ± 0.018	0.093 ± 0.015	0.083 ± 0.021	0.074 ± 0.015
5.00	0.074 ± 0.020	0.085 ± 0.016	0.071 ± 0.017	0.094 ± 0.019	0.087 ± 0.016	0.095 ± 0.020
5.25	0.079 ± 0.024	0.109 ± 0.041**	0.099 ± 0.018	0.114 ± 0.016	0.100 ± 0.032	0.102 ± 0.013
5.50	0.059 ± 0.023	0.079 ± 0.022	0.088 ± 0.033	0.090 ± 0.018	0.079 ± 0.028	0.079 ± 0.031
5.75	0.048 ± 0.020	0.070 ± 0.019*	0.059 ± 0.025	0.067 ± 0.026	0.057 ± 0.021	0.061 ± 0.009
6.00	0.066 ± 0.024	0.095 ± 0.028*	0.071 ± 0.025	0.083 ± 0.020	0.082 ± 0.032	0.083 ± 0.026
6.25	0.044 ± 0.023	0.065 ± 0.019	0.058 ± 0.032	0.072 ± 0.020	0.059 ± 0.030	0.071 ± 0.027
6.50	0.046 ± 0.022	0.062 ± 0.033	0.056 ± 0.025	0.063 ± 0.023	0.052 ± 0.022	0.064 ± 0.018
6.75	0.143 ± 0.026	0.164 ± 0.028	0.146 ± 0.042	0.153 ± 0.026	0.170 ± 0.049	0.142 ± 0.029
7.00	0.224 ± 0.040	0.232 ± 0.030	0.231 ± 0.041	0.236 ± 0.046	0.250 ± 0.050	0.237 ± 0.036
7.25	0.322 ± 0.055	0.328 ± 0.049	0.345 ± 0.066	0.366 ± 0.038	0.367 ± 0.083	0.355 ± 0.029
7.50	0.374 ± 0.058	0.359 ± 0.054	0.377 ± 0.078	0.434 ± 0.042^Δ^	0.434 ± 0.097^Δ^	0.393 ± 0.032
7.75	0.404 ± 0.066	0.408 ± 0.066	0.415 ± 0.074	0.470 ± 0.047	0.462 ± 0.104	0.441 ± 0.047
8.00	0.473 ± 0.075	0.457 ± 0.075	0.470 ± 0.084	0.540 ± 0.041^Δ^	0.536 ± 0.100^Δ^	0.496 ± 0.058
8.25	0.480 ± 0.080	0.474 ± 0.075	0.503 ± 0.087	0.565 ± 0.056^Δ^	0.572 ± 0.104^ΔΔ^	0.525 ± 0.049
8.50	0.494 ± 0.077	0.482 ± 0.072	0.489 ± 0.079	0.555 ± 0.048^Δ^	0.559 ± 0.106^Δ^	0.521 ± 0.046
8.75	0.571 ± 0.078	0.546 ± 0.088	0.582 ± 0.105	0.624 ± 0.076	0.633 ± 0.121^Δ^	0.581 ± 0.077
9.00	0.660 ± 0.100	0.635 ± 0.095	0.682 ± 0.105	0.741 ± 0.066^Δ^	0.741 ± 0.162^Δ^	0.686 ± 0.060
9.25	0.690 ± 0.099	0.671 ± 0.104	0.729 ± 0.114	0.800 ± 0.068^ΔΔ^	0.787 ± 0.152^Δ^	0.740 ± 0.070
9.50	0.750 ± 0.096	0.738 ± 0.114	0.783 ± 0.120	0.841 ± 0.051^Δ^	0.846 ± 0.147^Δ^	0.808 ± 0.070
9.75	0.809 ± 0.118	0.778 ± 0.117	0.856 ± 0.137	0.891 ± 0.080^Δ^	0.890 ± 0.163^Δ^	0.874 ± 0.069
10.00	0.846 ± 0.123	0.791 ± 0.113	0.891 ± 0.140	0.930 ± 0.102^Δ^	0.906 ± 0.168^Δ^	0.926 ± 0.074^Δ^
10.25	0.835 ± 0.135	0.791 ± 0.117	0.899 ± 0.136^Δ^	0.911 ± 0.086^Δ^	0.898 ± 0.146^Δ^	0.920 ± 0.086^Δ^
10.50	0.739 ± 0.140	0.676 ± 0.118	0.801 ± 0.121^Δ^	0.822 ± 0.081^ΔΔ^	0.785 ± 0.119^Δ^	0.824 ± 0.070^ΔΔ^
10.75	0.655 ± 0.121	0.600 ± 0.099	0.722 ± 0.104^ΔΔ^	0.698 ± 0.077^Δ^	0.693 ± 0.110^Δ^	0.739 ± 0.072^ΔΔ^
11.00	0.580 ± 0.115	0.551 ± 0.097	0.640 ± 0.100^Δ^	0.633 ± 0.045^Δ^	0.616 ± 0.100	0.645 ± 0.059^Δ^
11.25	0.548 ± 0.094	0.505 ± 0.081	0.590 ± 0.089^Δ^	0.585 ± 0.047^Δ^	0.564 ± 0.079	0.595 ± 0.067^Δ^
11.50	0.496 ± 0.090	0.456 ± 0.091	0.540 ± 0.082^Δ^	0.544 ± 0.041^Δ^	0.526 ± 0.083^Δ^	0.551 ± 0.048^ΔΔ^
11.75	0.433 ± 0.086	0.415 ± 0.086	0.469 ± 0.061	0.483 ± 0.071^Δ^	0.475 ± 0.081	0.491 ± 0.040^Δ^
12.00	0.407 ± 0.067	0.377 ± 0.067	0.428 ± 0.062	0.429 ± 0.048	0.432 ± 0.071^Δ^	0.439 ± 0.040^Δ^
12.25	0.410 ± 0.057	0.383 ± 0.065	0.422 ± 0.065	0.430 ± 0.039	0.432 ± 0.079	0.434 ± 0.039
12.50	0.387 ± 0.064	0.385 ± 0.057	0.405 ± 0.064	0.427 ± 0.044	0.419 ± 0.068	0.428 ± 0.036
12.75	0.360 ± 0.077	0.377 ± 0.060	0.399 ± 0.051	0.408 ± 0.042	0.407 ± 0.057	0.403 ± 0.037
13.00	0.362 ± 0.065	0.351 ± 0.075	0.387 ± 0.056	0.394 ± 0.031	0.379 ± 0.051	0.393 ± 0.033
13.25	0.462 ± 0.051	0.447 ± 0.072	0.473 ± 0.072	0.441 ± 0.058	0.452 ± 0.093	0.474 ± 0.059
13.50	0.587 ± 0.073	0.554 ± 0.071	0.595 ± 0.099	0.584 ± 0.064	0.586 ± 0.095	0.621 ± 0.076
13.75	0.590 ± 0.092	0.555 ± 0.092	0.629 ± 0.091	0.592 ± 0.091	0.582 ± 0.087	0.640 ± 0.060^Δ^
14.00	0.509 ± 0.113	0.454 ± 0.091	0.519 ± 0.072	0.534 ± 0.076^Δ^	0.513 ± 0.077	0.555 ± 0.047^ΔΔ^
14.25	0.426 ± 0.095	0.396 ± 0.078	0.427 ± 0.063	0.450 ± 0.079	0.440 ± 0.063	0.465 ± 0.035^Δ^
14.50	0.363 ± 0.076	0.351 ± 0.076	0.366 ± 0.053	0.380 ± 0.058	0.375 ± 0.050	0.390 ± 0.044
14.75	0.258 ± 0.072	0.255 ± 0.048	0.266 ± 0.050	0.267 ± 0.047	0.275 ± 0.052	0.268 ± 0.032
15.00	0.205 ± 0.058	0.217 ± 0.041	0.214 ± 0.043	0.219 ± 0.035	0.226 ± 0.030	0.222 ± 0.023
15.25	0.177 ± 0.050	0.185 ± 0.032	0.169 ± 0.032	0.172 ± 0.032	0.173 ± 0.035	0.180 ± 0.015
15.50	0.178 ± 0.033	0.196 ± 0.035	0.185 ± 0.032	0.179 ± 0.019	0.202 ± 0.043	0.190 ± 0.028
15.75	0.172 ± 0.037	0.193 ± 0.026	0.184 ± 0.044	0.180 ± 0.022	0.189 ± 0.033	0.180 ± 0.011
16.00	0.137 ± 0.038	0.158 ± 0.030	0.152 ± 0.027	0.139 ± 0.030	0.166 ± 0.031	0.146 ± 0.016

WL: wavelength, NC: normal control group, MC: model control group, HPM: herb-partition moxibustion, GPM: ginger-partition moxibustion, GLM: garlic-partition moxibustion, WM: warming moxibustion. The intensity of infrared radiation of the left-hand-side ST25 showed significant differences between normal rats and model rats (*P < 0.05, **P < 0.01). After moxibustion stimulations, the intensity of infrared radiation of ST25 in moxibustion groups were significantly different from those of the model groups (^Δ^ *P* < 0.05, ^ΔΔ^ *P* < 0.01).

**Table 4:** Intensity of infrared radiation of right-hand-side ST25 after different moxibustion treatments.

WL (*μ*m)	NC	MC	HPM	GPM	GLM	WM
1.50	−0.004 ± 0.031	0.009 ± 0.014	0.004 ± 0.022	0.006 ± 0.015	0.012 ± 0.020	0.005 ± 0.018
1.75	0.001 ± 0.022	0.021 ± 0.028*	−0.005 ± 0.015^ΔΔ^	0.015 ± 0.011	0.020 ± 0.022	0.003 ± 0.011^Δ^
2.00	−0.002 ± 0.024	0.007 ± 0.025	−0.003 ± 0.021	0.013 ± 0.021	0.009 ± 0.021	0.006 ± 0.020
2.25	0.005 ± 0.020	0.018 ± 0.026	−0.001 ± 0.019^Δ^	0.014 ± 0.017	0.011 ± 0.024	0.007 ± 0.019
2.50	0.005 ± 0.029	0.013 ± 0.027	−0.005 ± 0.011	0.009 ± 0.014	0.006 ± 0.020	0.004 ± 0.014
2.75	0.001 ± 0.022	0.022 ± 0.017**	0.002 ± 0.011^ΔΔ^	0.010 ± 0.011	0.010 ± 0.016	0.000 ± 0.008^ΔΔ^
3.00	0.002 ± 0.025	0.029 ± 0.021**	0.001 ± 0.023^ΔΔ^	0.003 ± 0.012^ΔΔ^	0.008 ± 0.017^Δ^	0.008 ± 0.014^Δ^
3.25	0.005 ± 0.023	0.016 ± 0.024	0.024 ± 0.024	0.011 ± 0.021	0.022 ± 0.017	0.014 ± 0.028
3.50	0.021 ± 0.028	0.036 ± 0.026	0.032 ± 0.030	0.016 ± 0.021	0.033 ± 0.021	0.025 ± 0.022
3.75	0.032 ± 0.031	0.039 ± 0.022	0.036 ± 0.022	0.039 ± 0.018	0.047 ± 0.022	0.041 ± 0.016
4.00	0.016 ± 0.015	0.038 ± 0.036*	0.039 ± 0.023	0.026 ± 0.018	0.040 ± 0.025	0.047 ± 0.010
4.25	0.048 ± 0.021	0.077 ± 0.041*	0.062 ± 0.030	0.053 ± 0.016	0.063 ± 0.029	0.062 ± 0.025
4.50	0.068 ± 0.021	0.074 ± 0.025	0.079 ± 0.025	0.083 ± 0.023	0.084 ± 0.031	0.074 ± 0.018
4.75	0.076 ± 0.029	0.096 ± 0.026*	0.085 ± 0.023	0.089 ± 0.014	0.091 ± 0.020	0.084 ± 0.012
5.00	0.094 ± 0.025	0.087 ± 0.019	0.090 ± 0.025	0.098 ± 0.018	0.097 ± 0.029	0.092 ± 0.015
5.25	0.106 ± 0.024	0.121 ± 0.039	0.109 ± 0.026	0.119 ± 0.010	0.113 ± 0.022	0.116 ± 0.019
5.50	0.082 ± 0.022	0.088 ± 0.030	0.093 ± 0.024	0.092 ± 0.015	0.089 ± 0.023	0.087 ± 0.019
5.75	0.057 ± 0.017	0.075 ± 0.029	0.074 ± 0.024	0.068 ± 0.030	0.063 ± 0.029	0.060 ± 0.013
6.00	0.082 ± 0.017	0.102 ± 0.024	0.088 ± 0.028	0.094 ± 0.016	0.093 ± 0.030	0.090 ± 0.020
6.25	0.068 ± 0.026	0.073 ± 0.034	0.072 ± 0.016	0.072 ± 0.016	0.071 ± 0.024	0.069 ± 0.024
6.50	0.059 ± 0.028	0.067 ± 0.035	0.068 ± 0.021	0.063 ± 0.022	0.057 ± 0.036	0.067 ± 0.017
6.75	0.147 ± 0.038	0.165 ± 0.039	0.177 ± 0.031	0.184 ± 0.025	0.169 ± 0.047	0.156 ± 0.020
7.00	0.230 ± 0.065	0.234 ± 0.042	0.270 ± 0.033	0.294 ± 0.036^ΔΔ^	0.256 ± 0.062	0.242 ± 0.026
7.25	0.341 ± 0.076	0.334 ± 0.053	0.401 ± 0.047^Δ^	0.420 ± 0.042^ΔΔ^	0.367 ± 0.082	0.352 ± 0.028
7.50	0.393 ± 0.064	0.378 ± 0.074	0.445 ± 0.048^Δ^	0.491 ± 0.060^ΔΔ^	0.421 ± 0.097	0.403 ± 0.037
7.75	0.428 ± 0.077	0.408 ± 0.078	0.482 ± 0.052^Δ^	0.524 ± 0.053^ΔΔ^	0.466 ± 0.094	0.442 ± 0.031
8.00	0.477 ± 0.087	0.472 ± 0.078	0.555 ± 0.062^Δ^	0.620 ± 0.062^ΔΔ^	0.541 ± 0.101	0.511 ± 0.058
8.25	0.509 ± 0.086	0.484 ± 0.088	0.591 ± 0.066^ΔΔ^	0.648 ± 0.070^ΔΔ^	0.565 ± 0.110^Δ^	0.536 ± 0.044
8.50	0.519 ± 0.095	0.491 ± 0.081	0.583 ± 0.063^Δ^	0.639 ± 0.077^ΔΔ^	0.559 ± 0.109	0.537 ± 0.041
8.75	0.590 ± 0.105	0.569 ± 0.088	0.678 ± 0.079^Δ^	0.720 ± 0.087^ΔΔ^	0.630 ± 0.133	0.609 ± 0.074
9.00	0.686 ± 0.122	0.657 ± 0.105	0.789 ± 0.074^ΔΔ^	0.850 ± 0.092^ΔΔ^	0.739 ± 0.132	0.711 ± 0.072
9.25	0.721 ± 0.134	0.698 ± 0.102	0.846 ± 0.088^ΔΔ^	0.914 ± 0.085^ΔΔ^	0.790 ± 0.153	0.761 ± 0.084
9.50	0.791 ± 0.133	0.772 ± 0.115	0.915 ± 0.087^Δ^	0.961 ± 0.087^ΔΔ^	0.828 ± 0.158	0.821 ± 0.083
9.75	0.850 ± 0.138	0.805 ± 0.124	0.984 ± 0.085^ΔΔ^	1.018 ± 0.071^ΔΔ^	0.893 ± 0.171	0.884 ± 0.094
10.00	0.892 ± 0.150	0.817 ± 0.131	1.027 ± 0.096^ΔΔ^	1.062 ± 0.072^ΔΔ^	0.905 ± 0.175	0.924 ± 0.088
10.25	0.874 ± 0.138	0.814 ± 0.134	1.030 ± 0.096^ΔΔ^	1.031 ± 0.073^ΔΔ^	0.866 ± 0.154	0.923 ± 0.087^Δ^
10.50	0.768 ± 0.117	0.711 ± 0.126	0.930 ± 0.089^ΔΔ^	0.908 ± 0.069^ΔΔ^	0.766 ± 0.130	0.822 ± 0.069^Δ^
10.75	0.679 ± 0.118	0.633 ± 0.115	0.834 ± 0.080^ΔΔ^	0.798 ± 0.055^ΔΔ^	0.677 ± 0.123	0.732 ± 0.075^Δ^
11.00	0.620 ± 0.110	0.573 ± 0.111	0.734 ± 0.061^ΔΔ^	0.712 ± 0.023^ΔΔ^	0.608 ± 0.112	0.655 ± 0.050^Δ^
11.25	0.556 ± 0.094	0.541 ± 0.107	0.674 ± 0.073^ΔΔ^	0.659 ± 0.031^ΔΔ^	0.551 ± 0.104	0.604 ± 0.055
11.50	0.501 ± 0.093	0.488 ± 0.095	0.619 ± 0.069^ΔΔ^	0.611 ± 0.031^ΔΔ^	0.515 ± 0.100	0.554 ± 0.045
11.75	0.448 ± 0.085	0.433 ± 0.092	0.548 ± 0.064^ΔΔ^	0.550 ± 0.032^ΔΔ^	0.457 ± 0.087	0.492 ± 0.033
12.00	0.427 ± 0.072	0.407 ± 0.069	0.496 ± 0.055^ΔΔ^	0.499 ± 0.023^ΔΔ^	0.431 ± 0.092	0.437 ± 0.029
12.25	0.430 ± 0.061	0.413 ± 0.070	0.490 ± 0.057^ΔΔ^	0.492 ± 0.028^ΔΔ^	0.419 ± 0.086	0.440 ± 0.030
12.50	0.420 ± 0.074	0.402 ± 0.065	0.475 ± 0.047^Δ^	0.483 ± 0.033^ΔΔ^	0.411 ± 0.087	0.431 ± 0.033
12.75	0.385 ± 0.070	0.380 ± 0.069	0.455 ± 0.052^ΔΔ^	0.470 ± 0.030^ΔΔ^	0.403 ± 0.072	0.410 ± 0.030
13.00	0.372 ± 0.067	0.365 ± 0.070	0.451 ± 0.049^ΔΔ^	0.449 ± 0.026^ΔΔ^	0.372 ± 0.076	0.398 ± 0.032
13.25	0.465 ± 0.092	0.465 ± 0.068	0.534 ± 0.051^Δ^	0.528 ± 0.051	0.448 ± 0.100	0.478 ± 0.050
13.50	0.605 ± 0.101	0.578 ± 0.091	0.692 ± 0.066^ΔΔ^	0.679 ± 0.044^Δ^	0.575 ± 0.112	0.617 ± 0.074
13.75	0.616 ± 0.106	0.580 ± 0.091	0.717 ± 0.074^ΔΔ^	0.704 ± 0.034^ΔΔ^	0.578 ± 0.115	0.640 ± 0.049
14.00	0.531 ± 0.100	0.473 ± 0.095	0.601 ± 0.069^ΔΔ^	0.617 ± 0.026^ΔΔ^	0.513 ± 0.105	0.555 ± 0.051^Δ^
14.25	0.467 ± 0.098	0.423 ± 0.086	0.503 ± 0.046^Δ^	0.528 ± 0.043^ΔΔ^	0.435 ± 0.084	0.467 ± 0.045
14.50	0.399 ± 0.083	0.375 ± 0.074	0.430 ± 0.040	0.451 ± 0.034^ΔΔ^	0.376 ± 0.076	0.395 ± 0.038
14.75	0.286 ± 0.066	0.267 ± 0.064	0.293 ± 0.035	0.314 ± 0.021^Δ^	0.274 ± 0.067	0.279 ± 0.025
15.00	0.227 ± 0.059	0.221 ± 0.050	0.238 ± 0.033	0.261 ± 0.032^Δ^	0.231 ± 0.035	0.223 ± 0.027
15.25	0.190 ± 0.043	0.196 ± 0.040	0.196 ± 0.025	0.203 ± 0.028	0.171 ± 0.045	0.189 ± 0.013
15.50	0.201 ± 0.041	0.201 ± 0.044	0.213 ± 0.028	0.218 ± 0.020	0.201 ± 0.050	0.191 ± 0.025
15.75	0.192 ± 0.029	0.208 ± 0.037	0.210 ± 0.020	0.209 ± 0.019	0.197 ± 0.045	0.189 ± 0.019
16.00	0.170 ± 0.026	0.164 ± 0.035	0.168 ± 0.029	0.174 ± 0.024	0.169 ± 0.035	0.157 ± 0.015

WL: wavelength, NC: normal control group, MC: model control group, HPM: herb-partition moxibustion, GPM: ginger-partition moxibustion, GLM: garlic-partition moxibustion, WM: warming moxibustion. The intensity of infrared radiation of right-hand-side ST25 showed significant differences between normal rats and model rats (*P < 0.05, **P < 0.01). After moxibustion stimulations, the intensity of infrared radiation of ST25 in moxibustion groups was significantly different from that of model groups (^Δ^ *P* < 0.05, ^ΔΔ^ *P* < 0.01).

## Abbreviations

ST25: Tianshu acupoint
NC: Normal control group
MC: Model control group
HPM: Herb-partition moxibustion
GPM: Ginger-partition moxibustion
GLM: Garlic-partition moxibustion
WM: Warming moxibustion
UC: Ulcerative colitis.
